# Experimental Study on Microwave-Based Curing Process with Thermal Expansion Pressure of PTFE for Manufacturing Carbon Fiber/Epoxy Composites

**DOI:** 10.3390/ma12223737

**Published:** 2019-11-13

**Authors:** Eu-Tteum Park, Youngheon Lee, Jeong Kim, Beom-Soo Kang, Woojin Song

**Affiliations:** 1 Department of Aerospace Engineering, Pusan National University, Busan 46241, Korea; bangsbang91@pusan.ac.kr (E.-T.P.); yhlee0323@pusan.ac.kr (Y.L.); greatkj@pusan.ac.kr (J.K.); bskang@pusan.ac.kr (B.-S.K.); 2 Graduate School of Convergence Science, Pusan National University, Busan 46241, Korea

**Keywords:** carbon fiber/epoxy composite, input power, microwave-based curing process, processing time, three-point bending test, uniaxial tensile test

## Abstract

Conventional composite curing incur high production costs because of their long processing times. In contrast, microwave curing process (MCP) can reduce the production costs because both the mold and the composite parts can be heated directly. In this study, a mold consisting of polytetrafluoroethylene (PTFE), quartz glass, and stainless steel clamps was manufactured to cure composite specimens of carbon fiber and epoxy resin. Flame test was conducted prior to the MCP to confirm whether the spark occurred in the mold and the composite prepreg. Uniaxial tensile tests and three-point bending tests were then conducted to obtain the mechanical properties of the composite specimens according to the input power and the processing time. The mechanical properties of the composite specimens fabricated by MCP were compared with those of composite specimens manufactured by PCF. The results show that MCP can cure the composite prepreg more rapidly than PCF and can attain comparable mechanical properties.

## 1. Introduction

Fiber-reinforced composites have shown outstanding specific strength and toughness compared with conventional metallic materials, and have therefore become widely used in the automotive and aircraft industries [1,2,3,4]. In addition, polymer nanocomposites such as carbon nano tube (CNT) and natural fiber-based composites have been researched to develop the multifunctional materials with a high added value in various areas such as aerospace, transport, energy, and health [5,6,7,8,9].

With increasing commercial deployment of the fiber-reinforced composites, various composite curing processes have been developed to improve the composite quality and production cycle. Autoclave processing cures composite prepreg by heating and pressurizing the air inside the autoclave chamber. Composite parts thus manufactured exhibit excellent structural performance compared with parts manufactured by conventional composite processes. However, autoclave processing requires a long time, and therefore incurs high production costs because of the time required to heat the air that then heats the composite prepreg by convection [10,11]. An alternative technique, prepreg compression forming (PCF), directly heats a metallic mold to reduce production costs [12,13]. Nonetheless, this technique can still require a long processing time depending on the thermal conductivity of the mold. In a recent development, high-pressure resin transfer molding (HP-RTM) has effectively reduced the processing time by injecting the resin at a high pressure and temperature [14,15]. However, the HP-RTM process incurs substantial energy costs in addition to the initial setup costs for the resin injection system [16].

A microwave-based curing process (MCP) is one solution for reducing the energy consumption and initial setup costs associated with a short process cycle [17,18,19,20,21]. The MCP heating mechanism operates through dipolar loss, conduction loss, hysteresis loss, and Eddy current loss [22]. For example, materials such as water, ceramics, and epoxy are composed of the dipoles. As these materials are subjected to a periodically changing electric field, the dipoles become unstable electrically and therefore vibrate. These vibrations induce internal inertia, elasticity, friction, adhesive force, etc. in the material, and heat flux associated with dipolar loss is generated in the material. In metallic materials or semiconductors, a magnetic field is generated when the free electrons in the material move in response to the periodically changing electric field. The induced magnetic field then generates reaction forces that push the free electrons in the opposite direction, and the repeated movements of these free electrons in the material generates heat flux associated with conduction loss because of the internal inertia, elasticity, friction, etc. In nickel, cobalt, and iron, the original magnetic state changes when microwave irradiate this material, and when irradiation ceases, the return to the original magnetic state is imperfect. This irreversible phenomenon causes some of the magnetic energy to be converted into heat energy, referred to as hysteresis loss. In the case of a conductor, an Eddy current is induced on the surface of the material when the penetration depth of the microwave is shorter than the thickness of the material, and the direction of the Eddy current is periodically changed by the magnetic field of the microwave. This change in the direction of the induced current results in some loss of magnetic energy in the form of heat energy. This generated heat energy is called the Eddy current loss. Because of these heating mechanisms, the MCP can generate volumetric heating in both the mold and the composite prepreg, reducing the processing time and production costs with saved energy consumption.

These advantages have stimulated interest in the MCP as a replacement for conventional curing processes. Joshi reported that the MCP for a composite prepreg based on carbon fiber reduced the processing time and energy consumption by factors of approximately 60% and 25%, respectively, compared with the autoclave process [23]. Li et al. compared the mechanical properties of composite specimens manufactured by the autoclave process and the MCP [24], reporting that the MCP produce composite specimens with mechanical properties comparable to those produced with the autoclave process and reduced the processing time and energy consumption by approximately 45% and 3%, respectively.

However, the MCP faces challenges to pressurizing the inside of the chamber to remove voids in the composite prepreg because the microwaves can damage the compressor and vacuum pump. Therefore, in this study, a mold based on polytetrafluoroethylene (PTFE) was manufactured to leverage PTFE’s thermal expansion pressure so that the MCP system would not require vulnerable components such as the compressor and the vacuum pump. When microwaves are irradiated inside the microwave oven, the electrode changes periodically like a dipole inside the mold because of the polarization. This repetitive change causes heat flux from the dipolar loss inside the mold, and the heat fluxes of the mold and the composite prepreg cause the thermal expansion of the PTFE plate. When the PTFE plate is retained inside the mold, the composite prepreg can be compressed without a compressor or vacuum pump.

To evaluate the proposed method, flame testing was conducted to determine whether or not a spark was generated on the mold and/or the composite prepreg, and then uniaxial tensile tests and three-point bending tests were conducted to investigate the effect of the input power and the processing time on the MCP. The mechanical properties of the composite specimens fabricated by the MCP were compared with the mechanical properties of composite specimens manufactured by conventional PCF.

## 2. Materials and Methods 

### 2.1. Design of Mold for Microwave-Based Curing Process (MCP)

#### 2.1.1. Manufacturing Mold

**Figure 1:**
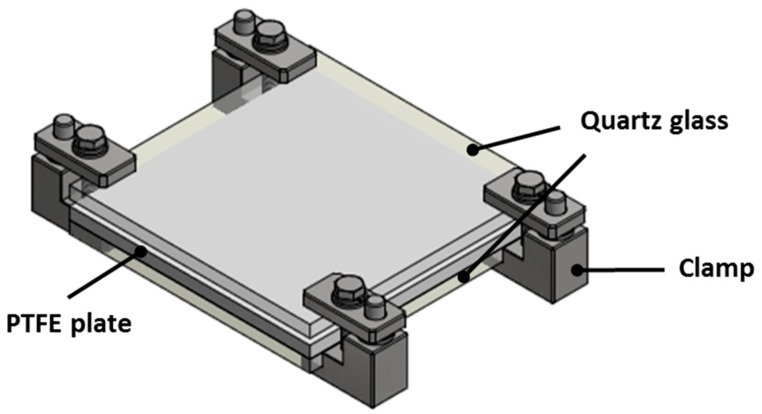
Configuration of mold for microwave-based curing process (MCP).

In this study, a commercial microwave oven, LG ML32W, was used to cure the composite prepreg, the frequency of which was 2.45 GHz. The width, length, and height of the oven were approximately 440, 235, and 310 mm, respectively. With reference to the size of the oven, the mold was manufactured as shown in Figure 1. Quartz glass was used to permit microwave transmission, and the PTFE plate was inserted between two quartz glass plates. The composite prepreg was inserted between the top quartz glass and the PTFE plate. In order to compress the composite prepreg, the mold was held by four stainless steel clamps.

#### 2.1.2. Flame Test

**Figure 2:**
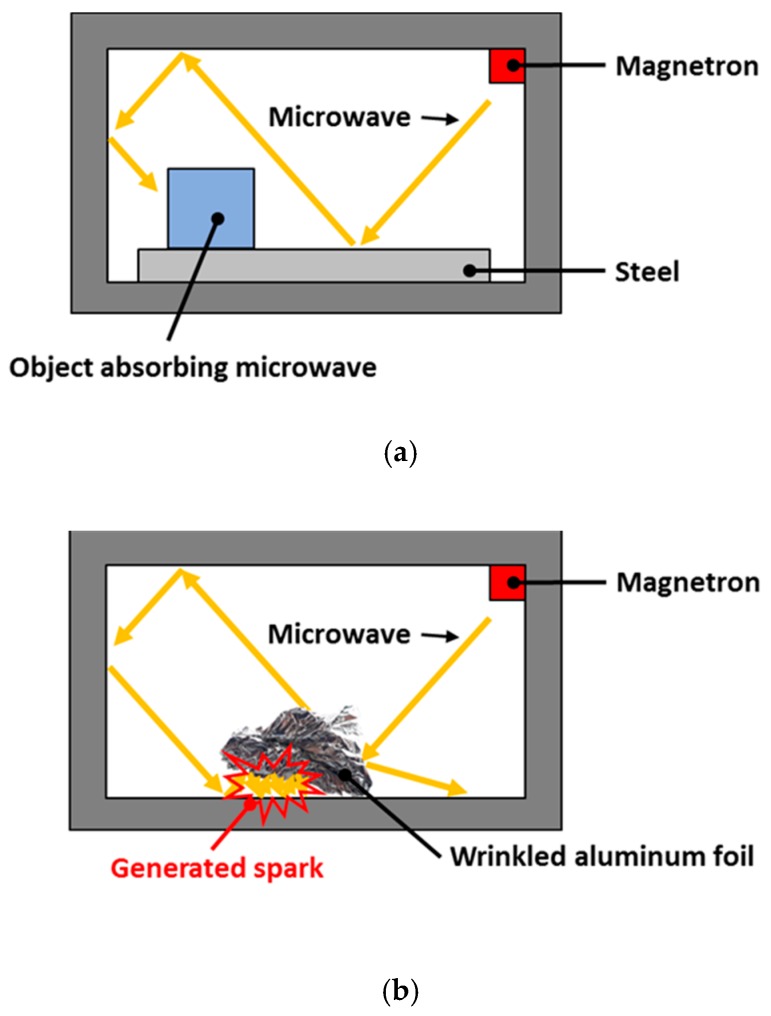
Spark generation principle: (**a**) flat metallic surface, (**b**) wrinkled aluminum foil.

When microwaves are irradiated, a spark may occur in the mold or at the edge of the composite prepreg. For example, most of the microwave is reflected from the flat surface of a metallic object as shown in Figure 2a. However, reflected microwaves can accumulate on wrinkled aluminum foil or on the edge of a fiber-based composite material as shown in Figure 2b. In these cases, the microwave energy is concentrated, and a spark is generated by the dielectric breakdown. Therefore, a flame test should be conducted to determine if a spark is generated in the mold and/or the composite prepreg.

**Figure 3:**
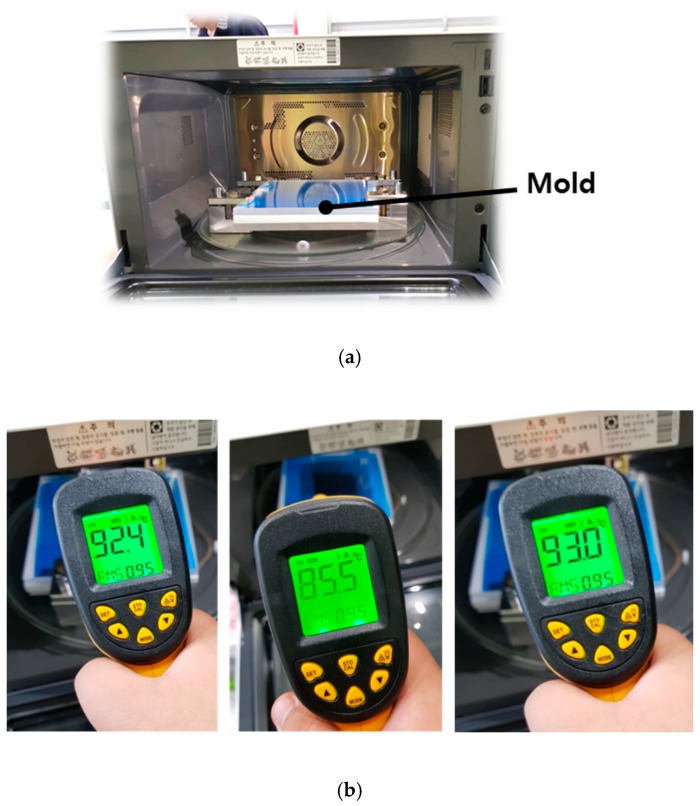
Flame test for mold and temperature measurement outside mold: (**a**) microwave oven with mold, (**b**) determination of temperature outside mold using an infrared radiation thermometer.

**Figure 4:**
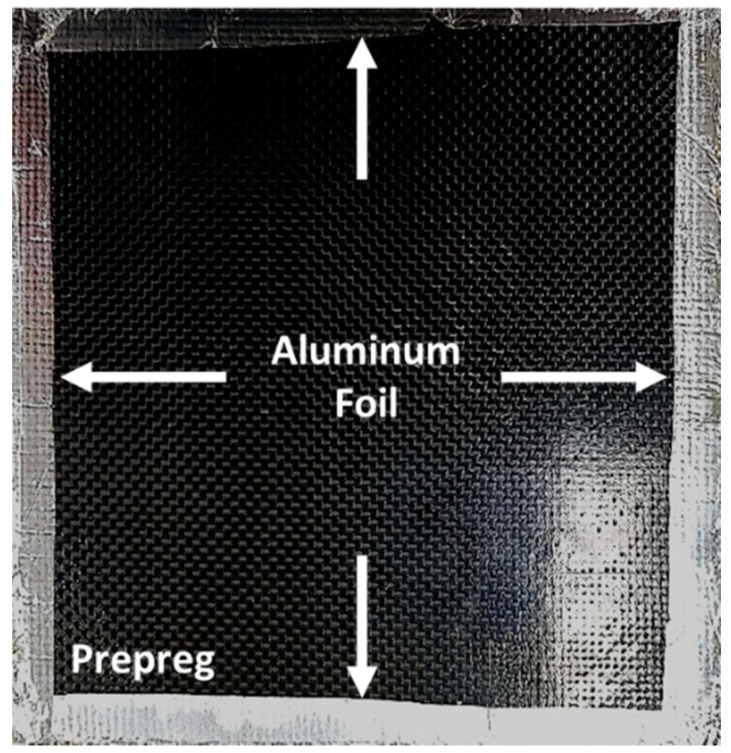
Carbon fiber/epoxy prepreg with aluminum foils at the prepreg edges.

In this study, a woven-type carbon fiber/epoxy prepreg (WSN 3k) was used. Its length and width were approximately 150 mm, and the thickness was approximately 0.21 mm. In order to manufacture a 2-mm-thick composite specimen, the composite prepregs were stacked by hand-layup method. The flame test was then conducted as shown in Figure 3a. These experiments applied 400 and 700 W of input power to confirm the generation of the spark, and the processing time was fixed at 30 min. When no spark was generated, the temperature on the mold was measured by an infrared radiation thermometer as shown in Figure 3b. Considering the curing temperature of the WSN 3k, the temperature was confirmed to reach approximately 130 °C. When the input power was 400 W, no sparks were generated on the mold or the composite prepreg, and the mold temperature was measured to be approximately 85~95 °C. However, when the input power was 700 W, sparks were generated on the edge of the composite prepreg. A thin aluminum foil was attached to the edge of the composite prepreg to prevent the dielectric breakdown, referencing Joshi [10]. The aluminum foil can delay the dielectric breakdown because aluminum has a sufficiently larger dielectric constant than the composite prepreg. The composite prepreg wrapped by the aluminum foil is shown in Figure 4, and each edge was punctured to allow excessive resin to drain from the composite prepreg when the sample was compressed by the thermal expansion pressure of the PTFE plate. With this treatment, no sparks were generated in the mold or the composite prepreg, and the temperature on the mold was measured to be approximately 110~140 °C. Thus, the flame test showed that the MCP can attain the cure temperature of the carbon fiber/epoxy prepreg.

### 2.2. Experimental Procedures

#### 2.2.1. Uniaxial Tensile Test

**Figure 5:**
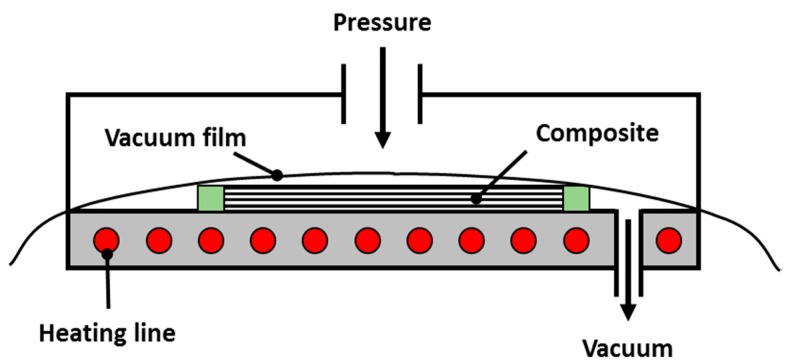
Configuration of prepreg compression forming (PCF) apparatus.

**Figure 6:**
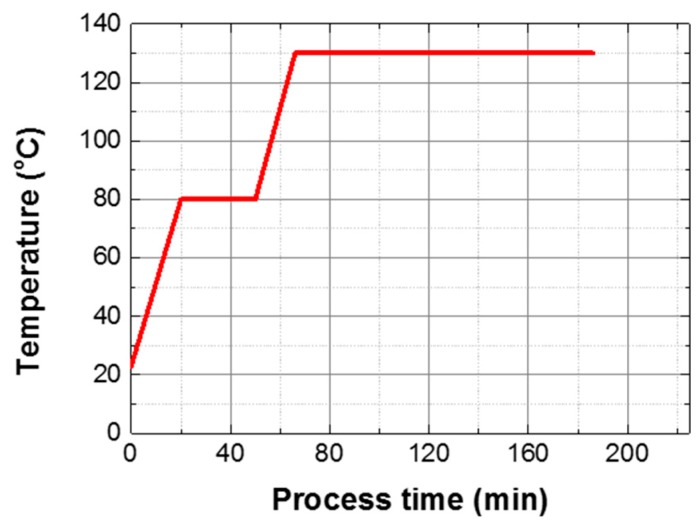
Curing cycle applied in PCF.

**Figure 7:**
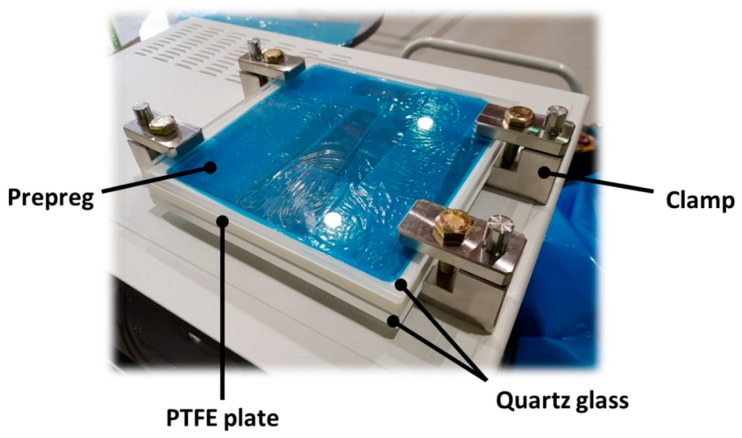
MCP mold containing carbon fiber/epoxy prepreg.

In this study, uniaxial tensile tests were carried out to obtain the Young’s modulus and the tensile strength of composite specimens manufactured by both PCF and MCP. First, prepreg wrapped with a release film and a vacuum film was placed inside the PCF chamber, as shown in Figure 5. The prepreg was then cured by a curing cycle that was obtained by the rule of thumb as shown in Figure 6. The PCF processing time was 185 min. During the curing cycle, a pressure of approximately 0.4 MPa and a vacuum pressure of 0.096 MPa were applied to the prepreg to discharge surplus resin. To test the MCP, the prepreg was inserted between the upper quartz glass plate and the PTFE plate after the release film was attached on the prepreg as shown in Figure 7, and the structural performance of the specimens was analyzed according to the input power and the processing time. The curing cases applied to respective MCP specimens are summarized in Table 1. In this chapter, three composite sheets were cured under each condition.

The specimens cured by PCF and MCP were cut into rectangles of approximately 175 mm long and 25 mm wide, in reference to ASTM D3039 [25]. The uniaxial tensile tests were conducted at a displacement rate of 2 mm/min. The ultimate strength was calculated as shown in Equation (1) using the load data obtained by the uniaxial tensile tests.

(1) $$\mathrm{Ultimate}\;\mathrm{strength}\;=\;\frac{F_{\max}}{wt}$$

Here, $F_{\max}$ is the maximum load, *w* is the width of the specimen, and *t* is the thickness of the specimen. The Young’s moduli of the specimens were obtained by linear curve fitting of the load data.

#### 2.2.2. Three-Point Bending Test

**Figure 8:**
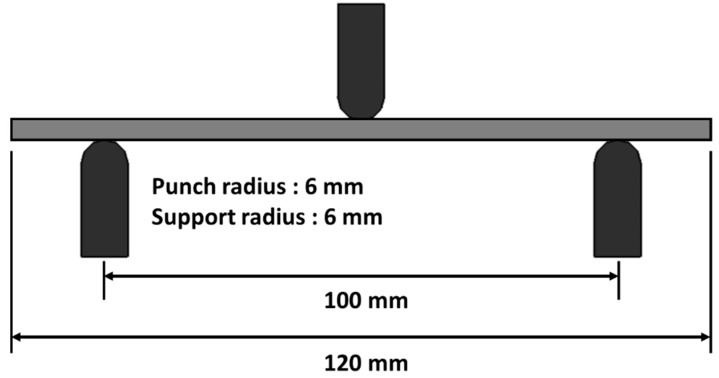
Schematic of three-point bending test.

Three-point bending tests were conducted to obtain the flexural strength and the flexural moduli of the composite specimens manufactured by PCF and MCP. MCP specimens were fabricated to test input powers of 200, 400, and 700 W, respectively, with processing times of 60 min each, as shown in Table 2. The specimens were cut into rectangles of approximately 120 mm long and 25 mm wide using a water-jet machine. As shown in Figure 8, one punch and two supports, each with a radius of approximately 6 mm, were used to conduct the tests. The spacing between the supports was approximately 100 mm. During the tests, the punch speed was approximately 2 mm/min. The flexural strain, flexural strength, and flexural modulus were calculated by the load data as expressed by Equations (2)–(4).

(2) $$\mathrm{Flexural}\;\mathrm{strain}\;=\;\frac{6Dd}{L^{2}}$$

(3) $$\mathrm{Flexural}\;\mathrm{strength}\;=\;\frac{3FL}{2bd^{2}}$$

(4) $$\mathrm{Flexural}\;\mathrm{modulus}\;=\;\frac{L^{3}\Delta }{4bd^{3}}$$

Here, *b* and *d* are the width and thickness of the specimen, respectively, *D* is the punch displacement, *F* is the reaction force of the punch, *L* is the interval between the supports, and Δ is the slope of the linear section in the reaction force versus punch displacement graph. In this chapter, three composite sheets were also cured under each condition.

## 3. Results and Discussions

### 3.1. Void Assessment

**Figure 9:**
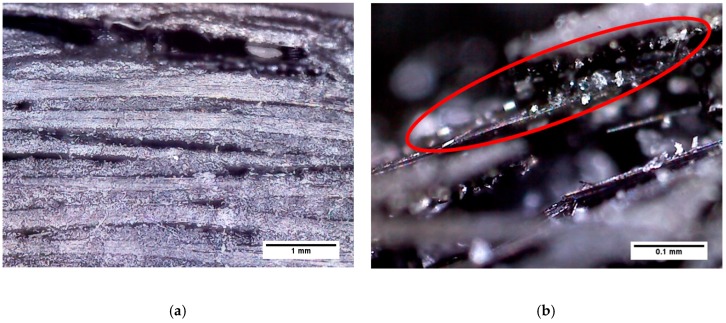

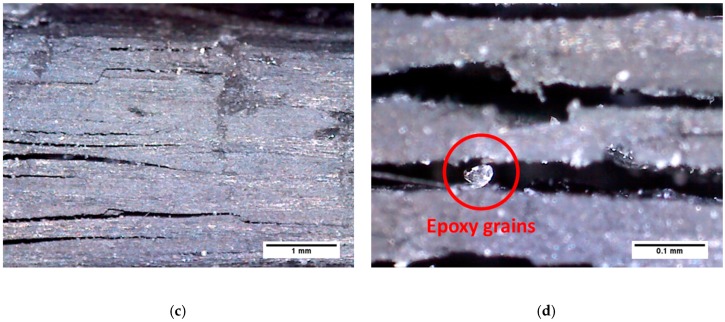
Microscope images of composite specimens with delamination.

**Figure 10:**
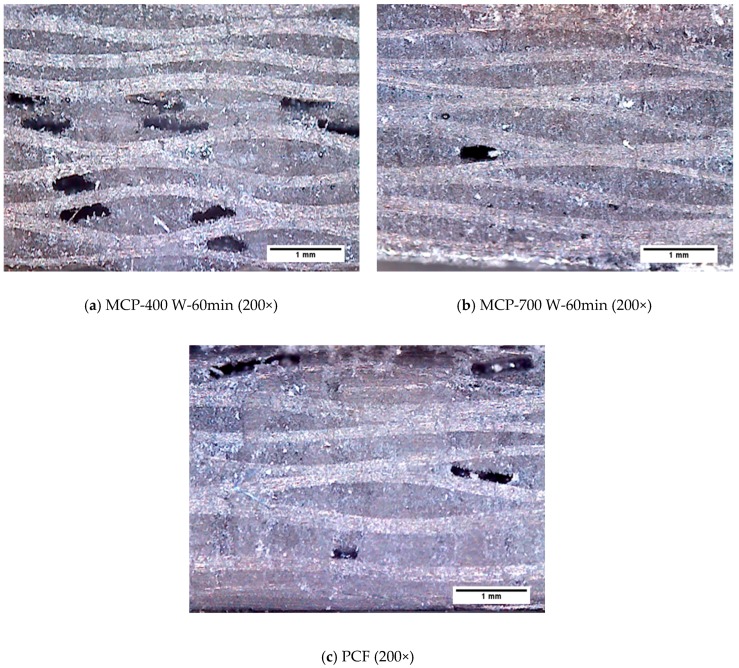
Microscope images of composite specimens without delamination.

Differences between the composite specimens fabricated by PCF and MCP were observed to assess the voids in the cross-sections of the specimens quantitively. Figure 9 shows microscopic images of the cross-sections of specimens cured by MCP, at 200x and 1000x magnification. As shown in Figure 9a,b, delamination occurred at 200 W of input power and 60 min of processing time because the epoxy was not perfectly cured. In particular, Figure 9b shows the epoxy grains that resulted from imperfect hardening. As shown in Figure 9c,d, the delamination also occurred at 700 W of input power and 30 min of processing time, and epoxy grains were also found on the cross-sections of these specimens. Therefore, when the processing time is short or the input power is insufficient, the epoxy is incompletely cured and epoxy grains are generated. This situation causes delamination in the composite materials. As shown in Figure 10, which presents images of MCP specimens that did not delaminate, the epoxy grains did not appear because the epoxy was cured perfectly. In addition, elongated oval pores can be observed in the cross-sections of these composite specimens. To quantify the pores of the specimens according to each condition, the total area of the pores per unit area were calculated using an image filtering technique, and the results are presented in Table 3. The composite specimens fabricated by PCF showed the lowest void ratio, and the composite specimens cured by MCP at 700 W of input power and 60 min of processing time showed an approximately 1.9% higher void ratio compared with the PCF specimens.

### 3.2. Effect of Input Power

**Figure 11:**
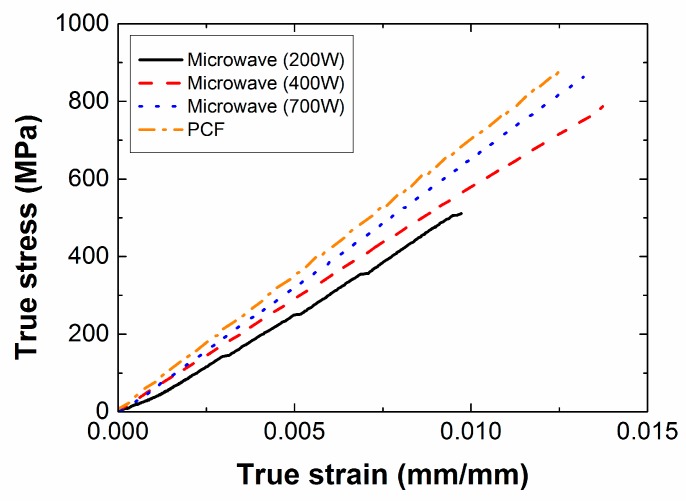
True stress-strain curves according to input power when processing time was 60 min.

**Figure 12:**
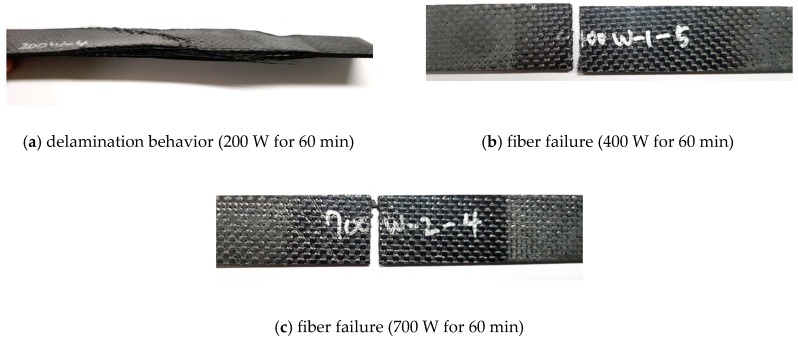
Failure modes of MCP specimens obtained from uniaxial tensile tests.

**Figure 13:**
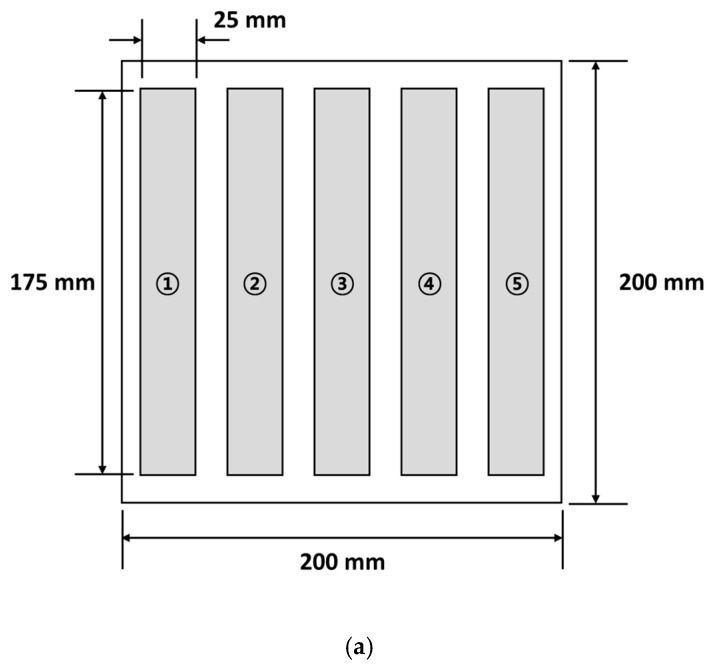

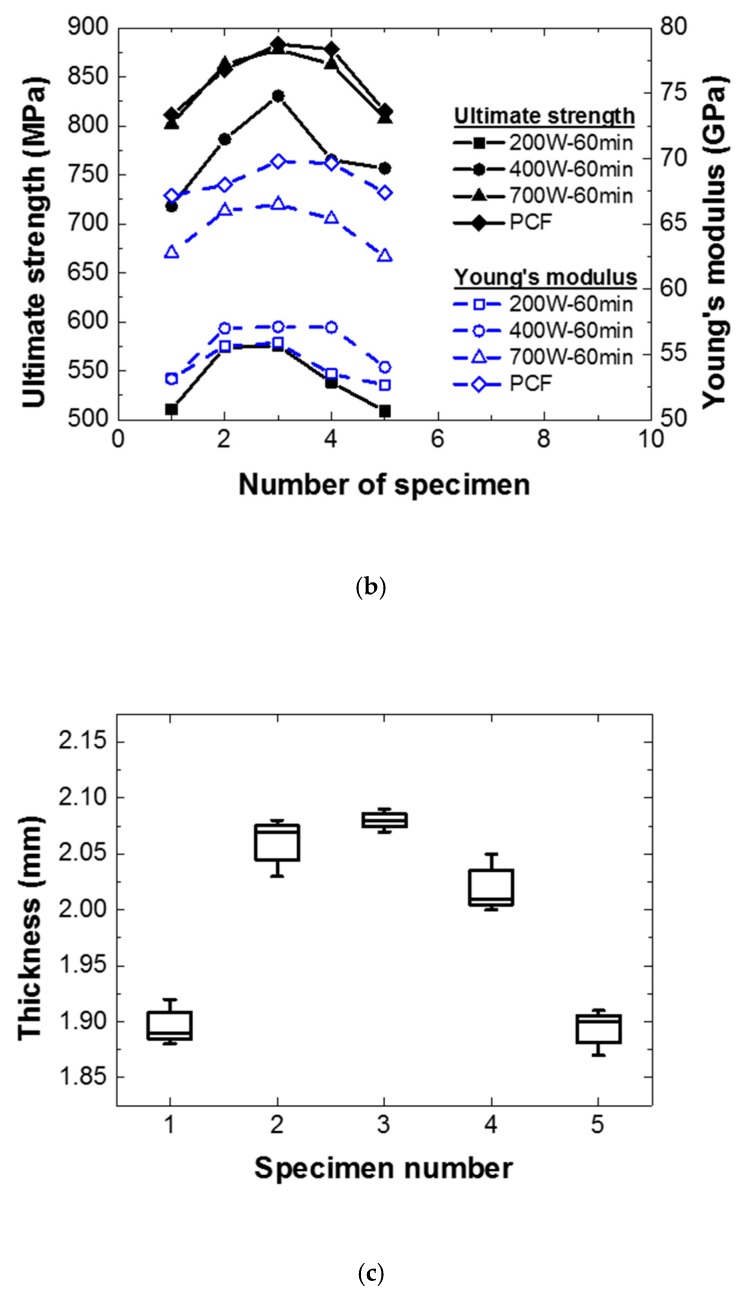
(**a**) Cutting location of specimens, (**b**) material properties according to cutting location, and (**c**) thickness of the MCP specimens (700 W for 60 min).

Figure 11 shows the stress-strain curves from the uniaxial tensile tests for PCF and MCP specimens cured with different input powers. The composite specimens cured by PCF showed the highest tensile strength and the composite specimens cured by MCP at 200 W of input power showed the lowest tensile strength. The mean value and standard deviations of each material property are summarized in Table 4 for further comparison. The composite specimens manufactured by PCF showed the highest tensile strength and Young’s modulus, and those cured by MCP at 200 W of input power and 60 min of processing time showed the lowest tensile strength and Young’s modulus. As shown in Figure 12a, delamination occurred before fracture in the MCP specimens cured with input power of 200 W because the carbon fiber did not transfer the load sufficiently to the other fibers owing to insufficient microwave energy. However, when the input power was 400 or 700 W, the carbon fibers were fractured without delamination as shown in Figure 12b,c. Compared with the PCF specimens, the tensile strength and Young’s modulus of the MCP specimens cured at 700 W of the input power, and 60 min of processing time showed relatively small differences of approximately 0.75% and 5.50%, respectively. As shown in Table 4, the standard deviations of the tensile strength and the Young’s modulus were approximately 4–6% of the mean value. To analyze the causes of these deviations in each specimen, their tensile strengths and Young’s moduli were compared, as shown in Figure 13b, according to the cutting location on the original composite sheet, which was a square with a side length of approximately 200 mm, as shown in Figure 13a. The specimens cut from the center of the original composite sheet exhibited the highest tensile strength and Young’s modulus, whereas the specimens cut from the edge of the original sheet exhibited the lowest tensile strength and Young’s modulus. This phenomenon was attributed to the fact that the surplus resin was discharged from the center to the edge by the pressure, and the fiber volume ratio at the center was therefore relatively higher than at the edge. Figure 13c shows the thickness of the MCP specimens cured with input power of 700 W and 60 min of processing time. It is noted that the thickness at the edge was relatively lower than at the center because the surplus resin was discharged by the pressure.

### 3.3. Effect of Processing Time

**Figure 14:**
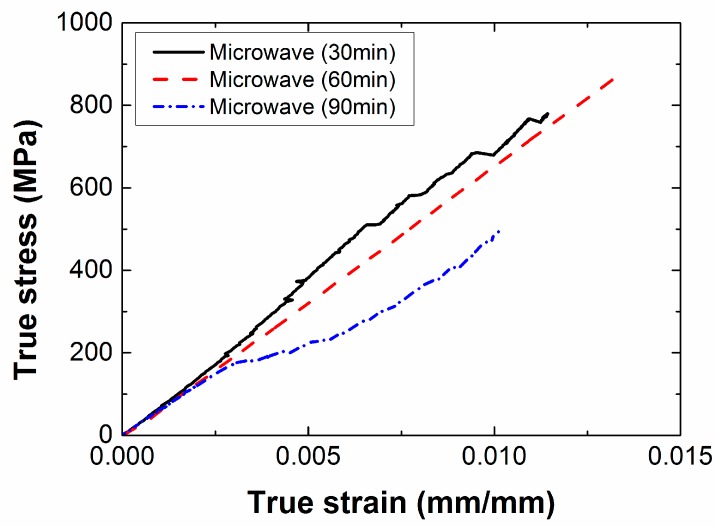
True stress-strain curves according to processing time when input power was 700 W.

The stress-strain curves obtained by the uniaxial tensile tests of specimens with different processing times are shown in Figure 14, and the tensile strength and Young’s modulus according to the processing time are summarized in Table 4. When the processing time was 60 min, the stress of the composite specimen linearly increased with the strain. When the processing time were 30 and 90 min, the stress did not linearly increase with the strain, and a relatively large noise was generated. When the processing time was 30 min, epoxy grains were generated because of incompletely cured epoxy on the internal surface of the specimens, which weakened the adhesion force between composite layers. This caused delamination to occur before the carbon fiber in the composite specimen fractured. When the processing time was 90 min, the internal temperature of the mold was excessively increased because of the long processing time. This decreased the tensile strength and the elongation of the composite specimens, and incurred some noise due to unbalanced adhesion between the epoxy and the carbon fiber.

### 3.4. Flexural Strength and Flexural Modulus

**Figure 15:**
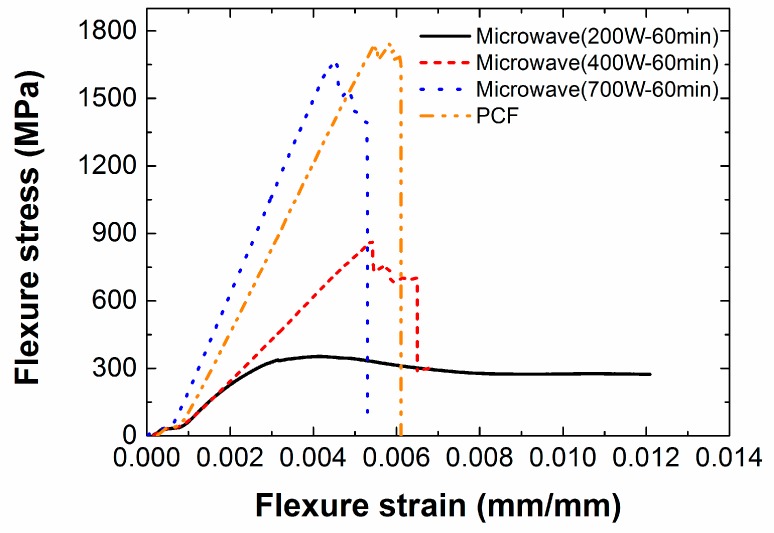
Flexural stress-strain curves obtained by three-point bending test.

**Figure 16:**
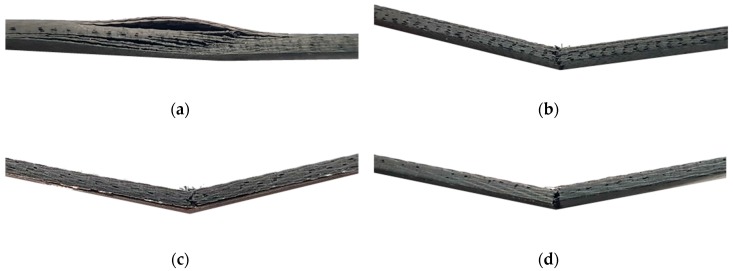
Failure modes of composite specimens on three-point bending test: (**a**) delamination behavior (MCP: 200 W for 60 min), (**b**) fiber failure (MCP: 400 W for 60 min), (**c**) fiber failure (MCP: 700 W for 60 min), (**d**) fiber failure (PCF).

Figure 15 shows the flexural strength diagram obtained by the three-point bending tests. When the input power was 200 W, the flexural stress increased nonlinearly with respect to the flexural strain. This nonlinearity can be explained by incomplete curing of the epoxy as mentioned in reference to the uniaxial tensile tests. Furthermore, in the MCP specimens fabricated at 200 W of input power, delamination occurred before the breakage of the carbon fiber because of incomplete curing of the epoxy as shown in Figure 16a. However, when the input power was 400 and 700 W, the carbon fiber fractured first, without delamination, as shown in Figure 16b–d.

**Figure 17:**
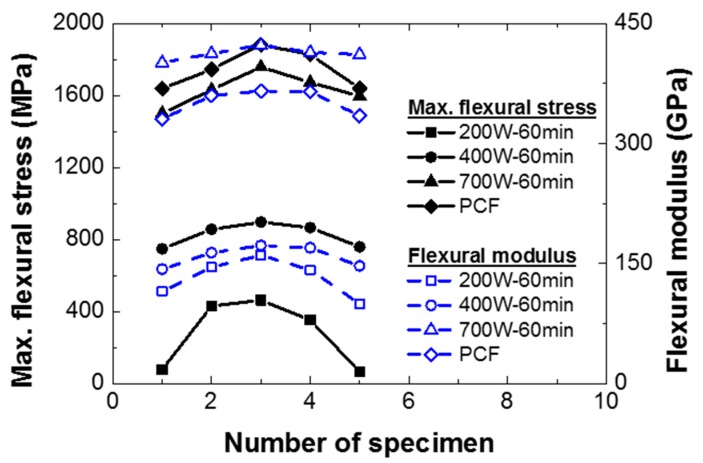
Material properties of composite specimens according to cutting location.

Table 4 shows the flexural strength and the flexural modulus of the composite specimens according to the input power. The composite specimens manufactured by PCF showed the highest flexural strength, approximately 6.55% higher than the composite specimens fabricated by MCP at 700 W of input power. For the flexural modulus, the composite specimens manufactured by MCP at 700 W of input power were approximately 17.44% higher than the composite specimens fabricated by PCF. These differences were attributed to inevitable errors in the experiments, and therefore the MCP specimens cured at 700 W of input power were considered to have acquired mechanical properties similar to those of the PCF specimens. In addition, MCP can cure the composite prepreg more efficiently than the PCF in terms of their processing times. As with the uniaxial tensile tests, the flexural strength and modulus of the MCP specimens cut from different locations on the original composite sheet were obtained, as shown in Figure 17. The composite specimens from the center of the original sheet showed the highest strength and modulus, and these properties decreased in specimens as their location moved toward the edge of the composite sheet.

## 4. Conclusions

The purpose of this study was to confirm the feasibility of the proposed MCP approach based on the thermal expansion pressure of the PTFE plate by comparing the mechanical properties of carbon fiber/epoxy composite specimens manufactured by PCF and MCP according to input power and processing time. The void ratios of the specimens were also determined by optical microscopy. The MCP specimens manufactured at 700 W of input power and 60 min of processing time showed void ratios similar to the PCF specimens. Subsequent uniaxial tensile tests and three-point bending tests of the specimens were conducted to compare their mechanical properties. For the MCP specimens, as the input power increased, the tensile strength and Young’s modulus also increased. The MCP specimens fabricated at 700 W of input power and 60 min of processing time had approximately 0.75% lower tensile strength and approximately 5.50% lower Young’s moduli than the PCF specimens. For the MCP specimens, when the input power and processing time were insufficient, the mechanical properties of the specimens decreased because of incompletely cured epoxy. When the processing time was excessively long, unstable deformation behavior was observed, and the mechanical properties were decreased because of weakened adhesion force between the epoxy and the carbon fiber. In the three-point bending tests, the MCP specimens manufactured at 700 W of input power and 60 min of processing time showed approximately 6.55% lower flexural strength than the PCF specimens. However, the flexural moduli of the MCP specimens were approximately 17.44% higher than those of the PCF specimens. As in the uniaxial tensile tests, when the input power to the MCP specimens was insufficient, their mechanical properties were decreased because of incompletely cured epoxy. In conclusion, the MCP specimens fabricated at 700 W of input power and 60 min of processing time showed mechanical properties similar to those of the PCF specimens, and MCP was therefore considered a more efficient alternative to PCF in terms of the processing time.

## Figures and Tables

**Table 1:** Uniaxial tensile test cases of MCP specimens.

Case	Input Power (W)	Processing Time (min)
1	200	60
2	400	60
3	700	30
4	700	60
5	700	90

**Table 2:** Three-point bending test cases of MCP specimens.

Case	Input Power (W)	Processing Time (min)
1	200	60
2	400	60
3	700	60

**Table 3:** Void areas and void ratios of composite specimens.

Curing process	Total Area (mm^2^)	Void Area (mm^2^)	Void Ratio (Void Area/Total Area)
Microwave (200 W/60 min)	18.7500	4.1213 ± 2.0317	0.3175
Microwave (400 W/60 min)		2.1660 ± 0.7439	0.1436
Microwave (700 W/60 min)		1.8623 ± 0.3325	0.0850
PCF		0.8190 ± 0.3749	0.0661

**Table 4:** Material properties according to input power and processing time.

Curing Process	Ultimate Strength (MPa)	Young’s Modulus (GPa)	Maximum Flexural Stress (MPa)	Flexural Modulus (GPa)
Microwave (200 W/60 min)	541.44 ± 32.40	54.18 ± 1.49	–	–
Microwave (400 W/60 min)	771.56 ± 41.32	55.67 ± 1.94	–	–
Microwave (700 W/60 min)	842.80 ± 35.26	64.64 ± 1.86	825.33 ± 67.25	159.12 ± 13.40
Microwave (700 W/30 min)	412.46 ± 78.67	54.18 ± 1.49	277.37 ± 192.67	132.45 ± 24.63
Microwave (700 W/90 min)	736.92 ± 30.17	65.60 ± 2.91	1632.23 ± 95.25	412.38 ± 7.89
PCF	849.20 ± 34.38	68.40 ± 1.25	1746.59 ± 109.81	351.15 ± 17.09

## References

[1] Erzen S., Ren Z., Anzel I. (2002). Analysis of FRP side-door impact beam. Proceedings of the 2nd IMechE Automobile Division Southern Centre Conference on Total Vehicle Technology.

[2] Abdullah M.R., Cantwell W.J. (2006). The impact resistance of polypropylene-based fibre-metal laminates. Compos. Sci. Technol..

[3] Takamatsu T., Matsumura T., Ogura N., Shimokawa T., Kakuta Y. (1999). Fatigue crack growth properties of a GLARE3–5/4 fiber/metal laminate. Eng. Fract. Mech..

[4] Vogelesang L.B., Vlot A. (2000). Development of fibre metal laminates for advanced aerospace structures. J. Mater. Process. Technol..

[5] Makaremi M., Pasbakhsh P., Cavallaro G., Lazzara G., Aw Y.K., Lee S.M., Milioto S. (2017). Effect of morphology and size of halloysite nanotubes on functional pectin bionanocomposites for food packaging applications. ACS Appl. Mater. Interfaces.

[6] Lvov Y., Abdullayev E. (2013). Functional polymer–clay nanotube composites with sustained release of chemical agents. Prog. Polym. Sci..

[7] Arrigo R., Teresi R., Gambarotti C., Parisi F., Lazzara G., Dintcheva N. (2018). Sonication-induced modification of carbon nanotubes: Effect on the rheological and thermo-oxidative behavior of polymer-based nanocomposites. Materials.

[8] Wang W., Yue X., Huang H., Wang C., Mo D., Wu Y., Xu Q., Zhou C., Zhu H., Zhang C. (2019). Electrical resistance prediction for functionalized multi-walled carbon nanotubes/epoxy resin composite gasket under thermal creep conditions. Materials.

[9] Kumar S., Nehra M., Dilbaghi N., Tankeshwar K., Kim K.H. (2018). Recent advances and remaining challenges for polymeric nanocomposites in healthcare applications. Prog. Polym. Sci..

[10] Centea T., Grunenfelder L.K., Nutt S.R. (2015). A review of out-of-autoclave prepregs—Material properties, process phenomena, and manufacturing considerations. Compos. Part A Appl. Sic. Manuf..

[11] Vita A., Castorani V., Germani M., Marconi M. (2019). Comparative life cycle assessment and cost analysis of autoclave and pressure bag molding for producing CFRP components. Int. J. Adv. Manuf. Technol..

[12] Wulfsberg J., Hermann A., Ziegmann G., Lonsdorfer G., Stöß N., Fette M. (2014). Combination of carbon fibre sheet moulding compound and prepreg compression moulding in aerospace industry. Procedia Eng..

[13] Lee J.M., Kim B.M., Ko D.C. (2019). Development of vacuum-assisted prepreg compression modling for production of automotive roof panels. Compos. Struct..

[14] Rondina F., Taddia S., Mazzocchetti L., Donati L., Minak G., Rosenberg P., Bedeschi A., Dolcini E. (2018). Development of full carbon wheels for sport cars with high-volume technology. Compos. Struct..

[15] Han B.J., Jeong Y.C., Kim C.M., Kim R.W., Kang M. (2019). Forming characteristics during the high-pressure resin transfer molding process for CFRP. Adv. Compos. Mater..

[16] Baskaran M., de Mendibil I.O., Sarrionandia M., Aurrekoetxea J., Acosta J., Argarate U., Chico D. Manufacturing cost comparison of RTM, HP-RTM and CRTM for an automotive roof. Proceedings of the 16th European Conference on Composite Materials.

[17] Lee W.I. (1984). Microwave curing of composites. J. Compos. Mater..

[18] Sung P.C., Chiu T.H., Chang S.C. (2014). Microwave curing of carbon nanotube/epoxy adhesives. Compos. Sci. Technol..

[19] Li N., Li Y., Hao X., Gao J. (2015). A comparative experiment for the analysis of microwave and thermal process induced strains of carbon fiber/bismaleimide composite materials. Compos. Sci. Technol..

[20] Li Y., Li N., Zhou J., Cheng Q. (2019). Microwave curing of multidirectional carbon fiber reinforced polymer composites. Compos. Struct..

[21] Xu X., Wang X., Cai Q., Wang X., Wei R., Du S. (2016). Improvement of the compressive strength of carbon fiber/epoxy composites via microwave curing. J. Mater. Sci. Technol..

[22] Mishra R.R., Sharma A.K. (2016). Microwave-material interaction phenomena: Heating mechanisms, challenges and opportunities in material processing. Compos. Part A Appl. Sic. Manuf..

[23] Joshi S.C., Bhudolia S.K. (2014). Microwave-thermal technique for energy and time efficient curing of carbon fiber reinforced polymer prepreg composites. J. Compos. Mater..

[24] Li N., Li Y., Jelonnek J., Link G., Gao J. (2017). A new process control method for microwave curing of carbon fibre reinforced composites in aerospace applications. Compos. Part B Eng..

[25] ASTM (2013). ASTM D3039-13-Standard Test Method for Tensile Properties of Polymer Matrix Composite Materials.

